# Taxonomy based on science is necessary for global conservation

**DOI:** 10.1371/journal.pbio.2005075

**Published:** 2018-03-14

**Authors:** Scott A. Thomson, Richard L. Pyle, Shane T. Ahyong, Miguel Alonso-Zarazaga, Joe Ammirati, Juan Francisco Araya, John S. Ascher, Tracy Lynn Audisio, Valter M. Azevedo-Santos, Nicolas Bailly, William J. Baker, Michael Balke, Maxwell V. L. Barclay, Russell L. Barrett, Ricardo C. Benine, James R. M. Bickerstaff, Patrice Bouchard, Roger Bour, Thierry Bourgoin, Christopher B. Boyko, Abraham S. H. Breure, Denis J. Brothers, James W. Byng, David Campbell, Luis M. P. Ceríaco, István Cernák, Pierfilippo Cerretti, Chih-Han Chang, Soowon Cho, Joshua M. Copus, Mark J. Costello, Andras Cseh, Csaba Csuzdi, Alastair Culham, Guillermo D’Elía, Cédric d’Udekem d’Acoz, Mikhail E. Daneliya, René Dekker, Edward C. Dickinson, Timothy A. Dickinson, Peter Paul van Dijk, Klaas-Douwe B. Dijkstra, Bálint Dima, Dmitry A. Dmitriev, Leni Duistermaat, John P. Dumbacher, Wolf L. Eiserhardt, Torbjørn Ekrem, Neal L. Evenhuis, Arnaud Faille, José L. Fernández-Triana, Emile Fiesler, Mark Fishbein, Barry G. Fordham, André V. L. Freitas, Natália R. Friol, Uwe Fritz, Tobias Frøslev, Vicki A. Funk, Stephen D. Gaimari, Guilherme S. T. Garbino, André R. S. Garraffoni, József Geml, Anthony C. Gill, Alan Gray, Felipe G. Grazziotin, Penelope Greenslade, Eliécer E. Gutiérrez, Mark S. Harvey, Cornelis J. Hazevoet, Kai He, Xiaolan He, Stephan Helfer, Kristofer M. Helgen, Anneke H. van Heteren, Francisco Hita Garcia, Norbert Holstein, Margit K. Horváth, Peter H. Hovenkamp, Wei Song Hwang, Jaakko Hyvönen, Melissa B. Islam, John B. Iverson, Michael A. Ivie, Zeehan Jaafar, Morgan D. Jackson, J. Pablo Jayat, Norman F. Johnson, Hinrich Kaiser, Bente B. Klitgård, Dániel G. Knapp, Jun-ichi Kojima, Urmas Kõljalg, Jenő Kontschán, Frank-Thorsten Krell, Irmgard Krisai-Greilhuber, Sven Kullander, Leonardo Latella, John E. Lattke, Valeria Lencioni, Gwilym P. Lewis, Marcos G. Lhano, Nathan K. Lujan, Jolanda A. Luksenburg, Jean Mariaux, Jader Marinho-Filho, Christopher J. Marshall, Jason F. Mate, Molly M. McDonough, Ellinor Michel, Vitor F. O. Miranda, Mircea-Dan Mitroiu, Jesús Molinari, Scott Monks, Abigail J. Moore, Ricardo Moratelli, Dávid Murányi, Takafumi Nakano, Svetlana Nikolaeva, John Noyes, Michael Ohl, Nora H. Oleas, Thomas Orrell, Barna Páll-Gergely, Thomas Pape, Viktor Papp, Lynne R. Parenti, David Patterson, Igor Ya. Pavlinov, Ronald H. Pine, Péter Poczai, Jefferson Prado, Divakaran Prathapan, Richard K. Rabeler, John E. Randall, Frank E. Rheindt, Anders G. J. Rhodin, Sara M. Rodríguez, D. Christopher Rogers, Fabio de O. Roque, Kevin C. Rowe, Luis A. Ruedas, Jorge Salazar-Bravo, Rodrigo B. Salvador, George Sangster, Carlos E. Sarmiento, Dmitry S. Schigel, Stefan Schmidt, Frederick W. Schueler, Hendrik Segers, Neil Snow, Pedro G. B. Souza-Dias, Riaan Stals, Soili Stenroos, R. Douglas Stone, Charles F. Sturm, Pavel Štys, Pablo Teta, Daniel C. Thomas, Robert M. Timm, Brian J. Tindall, Jonathan A. Todd, Dagmar Triebel, Antonio G. Valdecasas, Alfredo Vizzini, Maria S. Vorontsova, Jurriaan M. de Vos, Philipp Wagner, Les Watling, Alan Weakley, Francisco Welter-Schultes, Daniel Whitmore, Nicholas Wilding, Kipling Will, Jason Williams, Karen Wilson, Judith E. Winston, Wolfgang Wüster, Douglas Yanega, David K. Yeates, Hussam Zaher, Guanyang Zhang, Zhi-Qiang Zhang, Hong-Zhang Zhou

**Affiliations:** 1 Museu de Zoologia da Universidade de São Paulo, São Paulo, Brazil; 2 Chelonian Research Institute, Oviedo, Florida, United States of America; 3 Bernice Pauahi Bishop Museum, Hawai‘i, United States of America; 4 International Commission on Zoological Nomenclature, Singapore; 5 Department of Marine Invertebrates, Australian Museum, New South Wales, Australia; 6 School of Biological, Earth & Environmental Sciences, University of New South Wales, New South Wales, Australia; 7 Departamento de Biodiversidad y Biología Evolutiva, Museo Nacional de Ciencias Naturales (CSIC), Madrid, Spain; 8 Department of Biology, University of Washington, Seattle, Washington, United States of America; 9 Universidad de Atacama, Copiapó, Chile, and Programa de Doctorado en Sistemática y Biodiversidad, Facultad de Ciencias Naturales y Oceanográficas, Universidad de Concepción, Concepción, Chile; 10 Department of Biological Sciences, National University of Singapore, Singapore; 11 Biodiversity and Biocomplexity Unit, Okinawa Institute of Science and Technology, Kunigami District, Okinawa Prefecture, Japan; 12 Laboratório de Ictiologia, Departamento de Zoologia, IBB-UNESP Campus de Botucatu, Botucatu, São Paulo, Brazil; 13 FishBase Information and Research Group, IRRI Campus, Los Baños, Laguna, Philippines; 14 Comparative Plant and Fungal Biology Department, Royal Botanic Gardens, Kew, London, United Kingdom; 15 Zoologische Staatssammlung München, Staatliche Naturwissenschaftliche Sammlungen Bayerns, München, Germany; 16 Department of Life Sciences, Natural History Museum, London, United Kingdom; 17 Australian National Herbarium, Centre for Australian National Biodiversity Research, CSIRO National Research Collections Australia, Australian Capital Territory, Australia; 18 College of Medicine, Biology and Environment, Research School of Biology, Australian National University, Australian Capital Territory, Australia; 19 Hawkesbury Institute for the Environment, Western Sydney University, Penrith, New South Wales, Australia; 20 Canadian National Collection of Insects, Arachnids and Nematodes, Agriculture and Agri-Food Canada, Ontario, Canada; 21 Institut de Systématique, Evolution, Biodiversité, Muséum National d'Histoire Naturelle, Sorbonne Universités, Paris, France; 22 Division of Invertebrate Zoology, American Museum of Natural History, New York, United States of America; 23 Department of Biology, Hofstra University, New York, United States of America; 24 Naturalis Biodiversity Center, Leiden, the Netherlands; 25 Royal Belgian Institute of Natural Sciences, Brussels, Belgium; 26 School of Life Sciences, University of KwaZulu-Natal, Scottsville, South Africa; 27 Plant Gateway Ltd., Yorkshire, United Kingdom; 28 Department of Natural Sciences, Gardner-Webb University, North Carolina, United States of America; 29 Department of Biology, Villanova University, Pennsylvania, United States of America; 30 Potato Research Center, Georgikon Faculty, University of Pannonia, Keszthely, Hungary; 31 Dipartimento di Biologia e Biotecnologia 'Charles Darwin', Sapienza Università di Roma, Rome, Italy; 32 Department of Earth and Planetary Sciences, Johns Hopkins University, Maryland, United States of America; 33 Department of Environmental Science and Technology, University of Maryland, Maryland, United States of America; 34 Department of Plant Medicine, Chungbuk National University, Cheongju, Korea; 35 Hawai‘i Institute of Marine Biology, University of Hawai‘i, Hawai‘i, United States of America; 36 Institute of Marine Science, University of Auckland, Auckland, New Zealand; 37 Centre for Agricultural Research, Hungarian Academy of Sciences, Martonvásár, Hungary; 38 Department of Zoology, Eszterházy Károly University, Eger, Hungary; 39 Herbarium, School of Biological Sciences, University of Reading, Reading, United Kingdom; 40 Instituto de Ciencias Ambientales y Evolutivas, Facultad de Ciencias, Universidad Austral de Chile, Valdivia, Chile; 41 Department of Biosciences, University of Helsinki, Helsinki, Finland; 42 Ornithological Nomenclature Expert; 43 Green Plant Herbarium, Department of Natural History, Royal Ontario Museum, Toronto, Ontario, Canada; 44 Nomenclature Specialist, CITES Animals Committee; Turtle Conservancy, New York, United States of America; 45 Global Wildlife Conservation, Texas, United States of America; 46 Department of Plant Anatomy, Institute of Biology, Eötvös Loránd University, Budapest, Hungary; 47 Department of Biosciences (Plant Biology), Viikki Plant Science Centre, Helsinki, Finland; 48 Illinois Natural History Survey, Illinois, United States of America; 49 Institute of Biodiversity Science and Sustainability, California Academy of Sciences, San Francisco, California, United States of America; 50 Royal Botanic Gardens, Kew, London, United Kingdom; 51 NTNU University Museum, Norwegian University of Science and Technology, Trondheim, Norway; 52 Institut de Biologia Evolutiva, Universitat Pompeu Fabra, Barcelona, Spain; 53 Bioveyda Biodiversity Inventories and Research, California, United States of America; 54 Department of Plant Biology, Ecology & Evolution and Herbarium, Oklahoma State University, Oklahoma, United States of America; 55 Research School of Earth Sciences, Australian National University, Australian Capital Territory, Australia; 56 Departamento de Biologia Animal, Instituto de Biologia, Universidade Estadual de Campinas, São Paulo, Brazil; 57 Senckenberg Naturhistorische Sammlungen, Dresden, Germany; 58 Department of Biology, University of Copenhagen, Copenhagen, Denmark; 59 Department of Botany, Smithsonian Institution, National Museum of Natural History, Washington, DC, United States of America; 60 Plant Pest Diagnostics Branch, California Department of Food and Agriculture, California, United States of America; 61 PPG Zoologia, Departamento de Zoologia, Instituto de Ciências Biológicas, Universidade Federal de Minas Gerais, Minas Gerais, Brazil; 62 Macleay Museum, University of Sydney, New South Wales, Australia; 63 Centre for Ecology and Hydrology, Edinburgh Research Station, Scotland, United Kingdom; 64 Laboratório de Coleções Zoológicas, Instituto Butantan, São Paulo, SP, Brazil; 65 Environmental Management, School of Applied and Biomedical Science, Federation University, Victoria, Australia; 66 PPG Biodiversidade Animal, Universidade Federal de Santa Maria, Rio Grande do Sul, Brazil; 67 National Museum of Natural History, Smithsonian Institution, Washington, DC, United States of America; 68 Department of Terrestrial Zoology, Western Australian Museum, Western Australia, Australia; 69 Museu Nacional da História Natural e da Ciência, Universidade de Lisboa, Lisboa, Portugal; 70 The Kyoto University Museum, Kyoto University, Kyoto, Japan; 71 Finnish Museum of Natural History (Botany), University of Helsinki, Helsinki, Finland; 72 Royal Botanic Garden Edinburgh, Scotland, United Kingdom; 73 School of Biological Sciences and Environment Institute, University of Adelaide, South Australia, Australia; 74 Nees Institut für Biodiversität der Pflanzen, Universität Bonn, Bonn, Germany; 75 Department of Plant Science and Biotechnology, University of Pannonia, Keszthely, Hungary; 76 Lee Kong Chian Natural History Museum, National University of Singapore, Singapore; 77 Denver Botanic Gardens, Denver, Colorado, United States of America; 78 Department of Biology, Earlham College, Indiana, United States of America; 79 Montana Entomology Collection, Montana State University, Montana, United States of America; 80 School of Environmental Sciences, University of Guelph, Ontario, Canada; 81 Instituto de Ambiente de Montaña y Regiones Áridas, Universidad Nacional de Chilecito, La Rioja, Argentina; 82 Martha N. and John C. Moser Chair in Arthropod Systematics and Biological Diversity, The Ohio State University, Ohio, United States of America; 83 Department of Biology, Victor Valley College, Victorville, California, United States of America; 84 Identification and Naming Department, Royal Botanic Gardens, Kew, London, United Kingdom; 85 Natural History Laboratory, Faculty of Science, Ibaraki University, Mito, Japan; 86 Natural History Museum and Botanical Garden, University of Tartu, Tartu, Estonia; 87 Plant Protection Institute, Centre for Agricultural Research, Hungarian Academy of Sciences, Budapest, Hungary; 88 Department of Zoology, Denver Museum of Nature & Science, Denver, Colorado, United States of America; 89 Department of Systematic and Evolutionary Botany, Universität Wien, Wien, Austria; 90 FishBase, Department of Zoology, Swedish Museum of Natural History, Stockholm, Sweden; 91 Department of Zoology, Museo Civico di Storia Naturale of Verona, Verona, Italy; 92 Departamento de Zoologia, Universidade Federal do Paraná, Paraná, Brazil; 93 Sezione di Zoologia degli Invertebrati e Idrobiologia, MUSE-Museo delle Scienze di Trento, Trento, Italy; 94 Centro de Ciências Agrárias, Ambientais e Biológicas, Universidade Federal do Recôncavo da Bahia, Bahia, Brazil; 95 Department of Biological Sciences, University of Toronto Scarborough, Ontario, Canada; 96 Department of Environmental Science and Policy, George Mason University, Virginia, United States of America; 97 Natural History Museum of Geneva, Geneva, Switzerland; 98 Department of Genetics and Evolution, University of Geneva, Geneva, Switzerland; 99 Departamento de Zoologia, Universidade de Brasília, Distrito Federal, Brazil; 100 Department of Integrative Biology, Oregon State Arthropod Collection, Oregon State University, Oregon, United States of America; 101 Henares 16, Velilla de San Antonio, Madrid, Spain; 102 Department of Vertebrate Zoology, National Museum of Natural History, Smithsonian Institution, Washington, DC, United States of America; 103 International Committee on Bionomenclature, Braunschweig, Germany; 104 Universidade Estadual Paulista (Unesp), Faculdade de Ciências Agrárias e Veterinárias, Jaboticabal, Departamento de Biologia Aplicada à Agropecuária, São Paulo, Brazil; 105 Faculty of Biology, Alexandru Ioan Cuza University of Iași, Iași, Romania; 106 Departamento de Biología, Universidad de Los Andes, Mérida, Venezuela; 107 Laboratorio de Morfología Animal, Universidad Autónoma del Estado de Hidalgo, Hidalgo, México; 108 H.W. Manter Laboratory of Parasitology, University of Nebraska-Lincoln, Lincoln, Nebraska, United States of America; 109 Robert Bebb Herbarium (OKL), University of Oklahoma, Oklahoma Biological Survey, Norman, Oklahoma, United States of America; 110 Department of Microbiology and Plant Biology, Norman, Oklahoma, United States of America; 111 Fiocruz Mata Atlântica, Fundação Oswaldo Cruz, Rio de Janeiro, Brazil; 112 Department of Civil and Environmental Engineering, Ehime University, Matsuyama, Japan; 113 Department of Zoology, Hungarian Natural History Museum, Budapest, Hungary; 114 Department of Science Education, Hiroshima University, Higashihiroshima, Japan; 115 Department of Earth Sciences, Natural History Museum, London, United Kingdom; 116 Kazan Federal University, Kremlyovskaya, Russia; 117 Museum für Naturkunde, Leibniz-Institut für Evolutions-und Biodiversitätsforschung, Berlin, Germany; 118 Centro de Investigación de la Biodiversidad y Cambio Climático (BioCamb) e Ingeniería en Biodiversidad y Recursos Genéticos, Universidad Tecnológica Indoamérica, Quito, Ecuador; 119 Integrated Taxonomic Information System, National Museum of Natural History, Smithsonian Institution, Washington, DC, United States of America; 120 Natural History Museum of Denmark, Copenhagen, Denmark; 121 Department of Botany, Szent István University, Budapest, Hungary; 122 University of Sydney, New South Wales, Australia; 123 Zoological Museum of Lomonosov Moscow State University, Moscow, Russia; 124 Biodiversity Institute, University of Kansas, Kansas, United States of America; 125 CITES Scientific Authority, Finnish Museum of Natural History (Botany), University of Helsinki, Helsinki, Finland; 126 Instituto de Botânica, São Paulo, Brazil; 127 Department of Entomology, Kerala Agricultural University, Kerala, India; 128 University of Michigan Herbarium EEB, Ann Arbor, Michigan, United States of America; 129 Chelonian Research Foundation, Massachusetts, United States of America; 130 Turtle Conservancy, New York, United States of America; 131 Instituto de Ciencias Marinas y Limnológicas, Facultad de Ciencias, Universidad Austral de Chile, Valdivia, Chile; 132 Kansas Biological Survey and the Biodiversity Institute, University of Kansas, Kansas, United States of America; 133 Laboratório de Ecologia e Conservação, Universidade Federal do Mato Grosso do Sul, Mato Grosso do Sul, Brazil; 134 Sciences Department, Museums Victoria, Victoria, Australia; 135 Department of Biology and Museum of Vertebrate Biology, Portland State University, Oregon, United States of America; 136 Department of Biological Sciences, Texas Tech University, Texas, United States of America; 137 Staatliches Museum für Naturkunde Stuttgart, Stuttgart, Germany; 138 Department of Bioinformatics and Genetics, Swedish Museum of Natural History, Stockholm, Sweden; 139 Laboratorio de Sistemática y Biología Comparada de Insectos, Instituto de Ciencias Naturales, Universidad Nacional de Colombia, Bogotá, Colombia; 140 Global Biodiversity Information Facility, Copenhagen, Denmark; 141 Fragile Inheritance Natural History, Ontario, Canada; 142 National Focal Point to the Convention on Biological Diversity, Royal Belgian Institute of Natural Sciences, Brussels, Belgium; 143 Department of Biology, Pittsburg State University, Kansas, United States of America; 144 Departamento de Zoologia, Instituto de Biociências, Universidade de São Paulo, São Paulo, Brazil; 145 Agricultural Research Council, Plant Protection Research Institute, South African National Collection of Insects, Queenswood, South Africa; 146 Section of Mollusks, Carnegie Museum of Natural History, Pennsylvania, United States of America; 147 Department of Zoology, Faculty of Science, Charles University, Prague, Czech Republic; 148 Museo Argentino de Ciencias Naturales 'Bernardino Rivadavia', Buenos Aires, Argentina; 149 National Parks Board, Singapore Botanic Gardens, Singapore; 150 Department of Ecology and Evolutionary Biology, University of Kansas, Kansas, United States of America; 151 Judicial Commission on Prokaryote Nomenclature, London, United Kingdom; 152 Botanische Staatssammlung München and SNSB IT Center, Staatliche Naturwissenschaftliche Sammlungen Bayerns, München, Germany; 153 Permanent ICN Nomenclature Committee for Fungi, International Mycological Association; 154 Museo Nacional de Ciencias Naturales (CSIC), Madrid, Spain; 155 Dipartimento di Scienze della Vita e Biologia dei Sistemi, Università di Torino, Torino, Italy; 156 Istituto per la Protezione Sostenibile delle Piante sez. di Torino, CNR, Torino, Italy; 157 Department of Environmental Sciences, University of Basel, Basel, Switzerland; 158 Allwetterzoo Münster, Münster, Germany; 159 Department of Biology, University of Hawai'i at Manoa, Hawai‘i, United States of America; 160 UNC Herbarium (NCU), North Carolina Botanical Garden, University of North Carolina at Chapel Hill, Chapel Hill, North Carolina, United States of America; 161 Abteilung Morphologie und Systematik der Tiere und Zoologisches Museum, Universität Göttingen, Göttingen, Germany; 162 Unité Mixte de Recherche, Peuplements Végétaux et Bioaggresseurs en Milieu Tropical, Université de La Réunion, Ile de La Réunion, France; 163 Essig Museum of Entomology, University of California, Berkeley, Berkeley, California, United States of America; 164 General Committee for Nomenclature [for algae, fungi and plants], Royal Botanic Gardens and Domain Trust, New South Wales, Australia; 165 Smithsonian Marine Station, Florida, United States of America; 166 Molecular Ecology and Fisheries Genetics Lab, School of Biological Sciences, Bangor University, Bangor, United Kingdom; 167 Department of Entomology, Entomology Research Museum, University of California, Riverside, California, United States of America; 168 Australian National Insect Collection, CSIRO National Research Collections Australia, Australian Capital Territory, Australia; 169 School of Life Sciences, Biodiversity Knowledge Integration Center, Hasbrouck Insect Collection, Arizona State University, Arizona, United States of America; 170 Landcare Research, Auckland, New Zealand; 171 School of Biological Sciences, University of Auckland, Auckland, New Zealand; 172 Institute of Zoology, Chinese Academy of Sciences, Chaoyang District, P. R. China

Taxonomy is a scientific discipline that has provided the universal naming and classification system of biodiversity for centuries and continues effectively to accommodate new knowledge. A recent publication by Garnett and Christidis [1] expressed concerns regarding the difficulty that taxonomic changes represent for conservation efforts and proposed the establishment of a system to govern taxonomic changes. Their proposal to “restrict the freedom of taxonomic action” through governing subcommittees that would “review taxonomic papers for compliance” and their assertion that “the scientific community’s failure to govern taxonomy threatens the effectiveness of global efforts to halt biodiversity loss, damages the credibility of science, and is expensive to society” are flawed in many respects. They also assert that the lack of governance of taxonomy damages conservation efforts, harms the credibility of science, and is costly to society. Despite its fairly recent release, Garnett and Christidis' proposition has already been rejected by a number of colleagues [2,3,4,5,6,7,8]. Herein, we contribute to the conversation between taxonomists and conservation biologists aiming to clarify some misunderstandings and issues in the proposition by Garnett and Christidis.

Placing governance over the science of taxonomy blurs the distinction between taxonomy and nomenclature. Garnett and Christidis’s proposal is far-reaching but represents a narrow perspective of taxonomy, as utilized by conservation, and reflects an increasingly broad misunderstanding throughout biology of the scientific basis of taxonomy, formalized nomenclature, and the relationship between them. This trend may have resulted from the attenuation of instruction in taxonomic principles and, in particular, nomenclature at many universities, in part because of a shift in research priorities away from taxonomy.

Garnett and Christidis assert that an “assumption that species are fixed entities underpins every international agreement on biodiversity conservation.” This assumption demonstrates a fundamental misunderstanding of taxonomy and the evolving view of what species represent. The essential features of science include documenting natural patterns and processes, developing and testing hypotheses, and refining existing ideas and descriptions of nature based on new data and insights. Taxonomy, the science of recognizing and delimiting species, adheres to these fundamental principles. Discoveries of new organisms together with advances in methodology continue unabated, leading to a constant reevaluation of the boundaries between taxonomic entities. Species (and higher taxa) comprise related organisms that may be clustered together differently depending on which sets of criteria are emphasized. Hey et al. [9] acknowledge “the inherent ambiguity of species in nature” but point out that “species-related research and conservation efforts can proceed without suffering from, and without fear of, the ambiguity of species.” Through taxonomic research, our understanding of biodiversity and classifications of living organisms will continue to progress. Any system that restricts such progress runs counter to basic scientific principles, which rely on peer review and subsequent acceptance or rejection by the community, rather than third-party regulation. Thiele and Yeates [10] cautioned that such a system “could lead to authoritarianism and a stifling of innovative taxonomic viewpoints. No other hypothesis-driven field of science would accept such a straitjacket”.

Taxonomy and associated nomenclature are not without problems. Even with a common set of facts, alternative interpretations of how to classify organisms can lead to differing classifications. However, the science of taxonomy is increasingly rigorous, which can improve the foundation for targeted legislative action regarding species [11,12]. Taxonomic instability does not affect all taxonomic groups equally. Garnett and Christidis provide examples from mammals and birds, which collectively represent a small fraction (<1%) of known biodiversity [13]. These groups tend to be the subject of greater levels of taxonomic “fine-tuning”—but less so in bats and rodents, groups in which basic species discoveries frequently take place—leading to disproportionately more lumping, splitting, and nomenclatural issues. In contrast, taxonomists working on most other groups of organisms, with vastly greater diversity, are focused on the basic tasks of discovering, delimiting, and describing species, rather than rearranging classifications of taxa already described. In extreme cases, taxonomic instability results in what has become known as “taxonomic vandalism” [14,15], which usually involves self-published or non–peer-reviewed taxonomic works that unnecessarily disrupt taxonomy without a solid scientific foundation. Academic freedom, needed for scientific progress, may yield undesirable results. However, over some 250 years of taxonomy, the number of authors that would be considered taxonomic vandals is very small, and further improvements to the Codes of nomenclature may reduce the harm they do without impinging on science. Scientists have long worked to achieve a universal species concept and an accompanying set of operational criteria that could serve to define species limits across most, if not all, groups of organisms; however, this task remains incomplete for a number of legitimate reasons [16,17,18,19]. Rather than promoting the establishment of a system that would arbitrarily bias community acceptance or rejections of species-level taxonomic hypotheses, many avenues of work seem more likely to improve taxonomy and the sciences that depend on it, including the following: efforts to improve our definitions of what a species is, incorporating more taxonomists into committees of conservation organizations, and providing aid in campaigns aiming to secure funding for education and research in taxonomy, among others.

## Does taxonomy hamper conservation?

Garnett and Christidis “contend that the scientific community's failure to govern taxonomy threatens the effectiveness of global efforts to halt biodiversity loss, damages the credibility of science, and is expensive to society.” We disagree.

The authors claim that species-splitting provides an incentive to trophy hunters to target small populations, affects biodiversity tallies in ways that negatively impact conservation, and results in inordinately higher funding to oversplit taxonomic groups; but they provide no evidence to support these claims. If hunters target endangered species, then such societal developments should be challenged, rather than used as justification for changing the way in which science is conducted. They cite data in Evans et al. [20] to imply that different taxonomic approaches between birds and mammals could lead to disproportionate funding relative to genetic diversity, when in fact those data (Figure 6 therein) show that the number of species in a group is not correlated with funding (e.g., fishes comprise 11% of species protected under the United States Endangered Species Act but receive 61% of government funding).

How does taxonomic instability affect conservation? Morrison et al. [21] “found that changes in taxonomy do not have consistent and predictable impacts on conservation”; they also found that “splitting taxa may tend to increase protection, and name changes may have the least effect where they concern charismatic organisms.” In African ungulates, Gippoliti et al. [22] describe cases where conservation management based on the Biological Species Concept overlooks evolutionarily significant units (recognized with the Phylogenetic Species Concept), with negative consequences. The splitting of legally protected taxa may result in species not being included by name in conservation legislation or regulations, thereby losing legal protection. However, well-crafted legislation includes mechanisms to extend protection despite taxonomic changes; initiatives such as Convention on International Trade in Endangered Species (CITES) and the International Union for Conservation of Nature (IUCN) specialist groups already link taxonomy and its changes with conservation [23]. Garnett and Christidis assert that taxonomic instability negatively affects conservation. However, artificial stability arising from insufficient taxonomic work can be particularly detrimental to conservation, causing mistargeting of conservation funding by misrepresentation of population size and distribution with the flow-on effects to conservation status [11,24,25].

## More bureaucracy is not the answer

**Fig 1:**
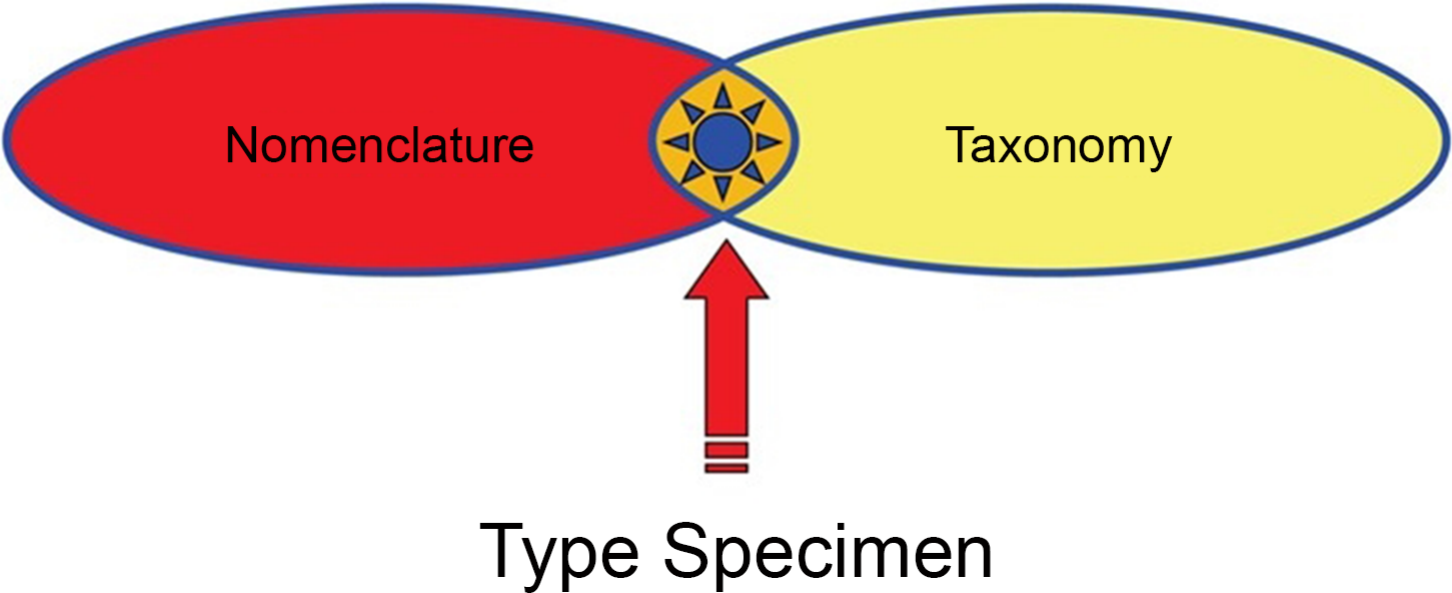
Nomenclature and taxonomy intersect objectively only at the type specimen, as designated through rules established by nomenclatural codes to anchor scientific names to the biological world.

The proposal by Garnett and Christidis for the International Union of Biological Sciences (IUBS) to create a process that “restrict[s] the freedom of taxonomic action” is not only flawed in terms of scientific integrity (as outlined above) but is also untenable in practice. Nomenclature regulates how names are used to communicate taxonomic hypotheses and is governed by rules (Codes) to ensure the least possible degree of ambiguity in the application of names. The relationship between taxonomy and nomenclature is illustrated in Fig 1. These Codes have been and continue to be refined into complex and intricate legal systems (the *International Code of Zoological Nomenclature* consists of 90 articles with more than 600 subsections). A system that endeavors to impose similar controls over taxon concepts would likely be vastly more complex than, and in conflict with, the Codes. It is for good reason that the major Codes explicitly avoid interfering with taxonomic freedom.

In addition, such a system raises many questions. Would it limit the kinds of characters used to assert taxonomically important distinctions, or be biased in favor of one class of characters (e.g., molecular versus morphological), when these cannot be equated across different taxa? How would new knowledge be incorporated? Would it favor one particular species concept for all organisms (and if so, which one)? Would newly discovered species automatically be acknowledged as legitimate new taxa or would they need to be approved before being considered valid? How often would the approved species lists be updated? Taking into account the vanishing taxonomic expertise, who would do this, and who would fund it? Can we afford to draw limited resources away from vital efforts to describe and catalogue biodiversity? There is already a scientific process to deal with updating taxonomy; “taxonomic revisions” carefully review all knowledge on a taxonomic group and may propose alternative classifications and relationships to accommodate new knowledge. These are peer-reviewed, published, and up to the community to accept or reject with further research. Furthermore, given that hundreds of thousands of species remain to be discovered, and that about 18,000 new species are described and named every year [26], adding layers of bureaucracy to this process would be both impractical and expensive. The governing structure proposed by Garnett and Christidis would need to include this peer review, consultation, and publication process regularly to reflect new knowledge. Therefore, it would add, and possibly duplicate, existing practice.

The products of taxonomic research underpin all biological research, but the proposal by Garnett and Christidis would regulate taxonomy primarily in the context of conservation. This has important potential ramifications because any supervisory body would implicitly have the power to direct, through its actions and judgments, the lumping or splitting of taxa according to conservation, economic significance, or political agendas to affect resource streams directed to those taxa. The process would also be vulnerable to conflicting pressures from advocacy groups in many areas, including conservation, trade, bioprospecting, and particularly politics. Even within birds, one of the groups that exemplify the problem that the proposal seeks to solve, taxonomic committees for managing taxa have had a mixed track record [27].

Certainly, there are many ways taxonomists can improve the value and impact of their research to conservation biology and other biological disciplines, such as explicitly citing the species concept employed in new taxonomic descriptions and including information on distributions, ecology, conservation status, and potential threats. Better and more modern approaches to organizing scientific names of organisms could also be expanded. In addition to overseeing the Codes of nomenclature, IUBS supports the International Committee on Bionomenclature (ICB) to promote harmony among the different Codes as nomenclature becomes increasingly digital. The development of online nomenclatural registration and indexing systems (e.g., the International Plant Names Index, ZooBank, various mycological registries, List of Prokaryotic Names with Standing in Nomenclature) offer improved access to nomenclatural information. These help avoid perpetuation of errors in the literature and thus increase stability and decrease ambiguity of taxon names.

Improvements are not limited to the Codes. Efforts such as the Catalogue of Life, with its numerous contributors and broad spectrum of users, already provide a valuable service for many taxonomic groups in asserting a reference classification and set of species concepts covering all life. This illustrates the potential for building a robust framework for a stable taxonomy to serve those initiatives that benefit from such stability, including conservation. These efforts can be improved by filling the existing gaps in taxa, training new taxonomists, improving the quality of information included for certain groups (e.g., distribution, conservation status), and by incorporating systems that track changes in both taxon names and circumscriptions through mapping of taxonomic concepts [28].

Dynamic taxonomy reflects the scientific nature and progress of the discipline. Artificially and arbitrarily constraining taxonomy through the system proposed by Garnett and Christidis would damage scientific credibility far more severely than misperceptions about the taxonomic process. “Absolute stability of taxonomic concepts—and nomenclature—would hinder scientific progress rather than promote it” [29].

## Conservation is crucial

The dynamic nature of taxonomic progress may be at odds with some aspects of conservation legislation, resulting, in part, from a mutual misunderstanding of the fundamental processes involved with both taxonomy and conservation. We advocate a solution that allows input, collaboration, and cooperation, from both conservation biologists and taxonomists, with a multidisciplinary approach towards a new framework for legislation that does not rely on the false premise that species are “fixed entities”. The development of “best practices” by both conservation biologists and taxonomists working together could avoid many unnecessary problems when using taxon names to represent vulnerable biological units in nature, thereby improving the effectiveness of their protection without impeding scientific progress.

Rather than redefine how one of the core disciplines of biological sciences is conducted, a more effective approach is to redefine how conservation legislation is enacted and implemented. The process of changing legislation requires acts of governments, which can take years to accomplish. However, fundamentally altering a system of classifying nature that has successfully endured more than two and a half centuries would have many detrimental consequences. Most of the problems for conservation resulting from the dynamic taxonomic process could be avoided entirely if future conservation legislation followed the lead of existing international conventions by explicitly referencing the specific taxon concept implied by a name, that is, by citing the original species description or a recent scholarly taxonomic treatment. Taxonomists and conservation biologists should join forces to promote effective legislative mechanisms to deal with a changing taxonomy rather than engage in infighting about the proper way to do taxonomy. This is exemplified by CITES, which adopts standard nomenclatural references [23] to define species or taxonomic groups and which periodically revises the adopted standards in response to evolving taxonomic consensus.

Many have argued that conservation legislation should focus on protecting entire ecosystems rather than rely on enumerated lists of species (e.g., [30]). While this approach requires a solid taxonomic foundation to characterize the ecosystems in question, the legislation itself would be insulated from specific changes to taxon names and concepts. In cases in which legislation includes specific taxa by name, such as harvesting or endangered species regulations, it should make the intended taxonomic concepts clear with reference to published treatments. That will allow unambiguous understanding even if the nomenclature and classification change because of taxonomic advances.

The critical importance of taxonomy and the taxonomic process in the global quest to mitigate biodiversity loss cannot be overemphasized. Without a robust taxonomic paradigm that is based on science and unconstrained by unnecessary and counterproductive bureaucracy, conservation efforts will ultimately suffer, potentially leading to devastating and irreversible impacts on global biodiversity.

## Abbreviations

CITES: Convention on International Trade in Endangered Species
ICB: International Committee on Bionomenclature
IUBS: International Union of Biological Sciences
IUCN: International Union for Conservation of Nature
